# Charting the perfect storm: emerging biological interfaces between stress and stroke

**DOI:** 10.1007/s00406-017-0794-x

**Published:** 2017-04-09

**Authors:** G. Kronenberg, J. Schöner, C. Nolte, A. Heinz, M. Endres, Karen Gertz

**Affiliations:** 1 Klinik und Poliklinik für Psychiatrie und Psychotherapie, Universitätsmedizin Rostock, Rostock, Germany; 2 Klinik und Poliklinik für Psychiatrie und Psychotherapie, Charité Universitätsmedizin Berlin, Charité Campus Mitte, Berlin, Germany; 3 Klinik und Poliklinik für Neurologie, Charité Universitätsmedizin Berlin, Charitéplatz 1, 10117 Berlin, Germany; 4 Center for Stroke Research Berlin (CSB), Charité Universitätsmedizin Berlin, Berlin, Germany; 5 German Center for Neurodegenerative Diseases (DZNE), Berlin, Germany; 6 German Center for Cardiovascular Research (DZHK), Berlin, Germany

**Keywords:** Depression, FKBP5, Heart rate, Psychosocial stress, Stroke, Telomerase

## Abstract

A growing body of evidence demonstrates that psychosocial stress is an important and often underestimated risk factor for cardiovascular disease such as myocardial infarction and stroke. In this article, we map out major biological interfaces between stress, stress-related psychiatric disorders, and stroke, placing special emphasis on the fact that stress and psychiatric disorders may be both cause and consequence of cardiovascular disease. Apart from high-risk lifestyle habits such as smoking and lack of exercise, neuroendocrine dysregulation, alterations of the hemostatic system, increased oxidative stress, and inflammatory changes have been implicated in stress-related endothelial dysfunction. Heart rate provides another useful and easily available measure that reflects the complex interplay of vascular morbidity and psychological distress. Importantly, heart rate is emerging as a valuable predictor of stroke outcome and, possibly, even a target for therapeutic intervention. Furthermore, we review recent findings highlighting the role of FK506-binding protein 51 (FKBP5), a co-chaperone of the glucocorticoid receptor, and of perturbations in telomere maintenance, as potential mediators between stress and vascular morbidity. Finally, psychiatric sequelae of cardiovascular events such as post-stroke depression or posttraumatic stress disorder are highly prevalent and may, in turn, exert far-reaching effects on recovery and outcome, quality of life, recurrent ischemic events, medication adherence, and mortality.

## Stress and cardiovascular disease (CVD)

Most soccer fans, kicking back in a comfortable recliner and looking forward to watching an exciting match, would be in stitches if they were told that, by watching soccer on television, they are indulging a perilous pastime. Notwithstanding, a Bavarian team of researchers found just that—that the incidence of cardiovascular events on days of suspenseful games during the 2006 FIFA World Cup in Munich was more than doubled [1]. The authors suggest that the thrill of an action-packed game triggered acute coronary syndromes, especially in men with pre-existing coronary heart disease (CHD) [1]. Whereas diabetes, obesity, hypertension, dyslipidemia, and smoking are nowadays firmly established as risk factors for myocardial infarction (MI) and stroke, the link between stress and cardiovascular disease (CVD) remains underrated by both clinicians and patients.

CVD is the leading cause of death worldwide, accounting for nearly one-third of all deaths in the United States in 2013 [2]. CVD imposes a tremendous economic burden, with direct and indirect costs amounting to $316.6 billion annually in North America. Within CVD, stroke caused 6.5 million deaths globally in 2013, making it the second leading cause of death on the planet [2]. Considering the high prevalence of CVD alongside the attendant extraordinary human and economic costs, the identification and prevention of risk factors is an area of pressing clinical concern.

The Framingham cohort study was among the first to document the importance of psychosocial factors in the etiopathogenesis of coronary heart disease (CHD) [3], indicating that certain behavioral patterns (Framingham Type A behavior, e.g., impatience, hostility, and sense of time urgency) and anxiety are associated with a higher risk of CHD. Until fairly recently, research on the association between psychosocial risk factors and CVD mainly focused on the economically advanced countries in Europe and North America. However, the relationship between negative psychosocial factors and cardiovascular disease holds across a wide range of ethnic, sociodemographic, and cultural groups: The cross-national INTERHEART study, which investigated 11,119 patients from all continents, confirmed that psychosocial stress increases the risk of acute MI in all ethnic, age, and gender groups [4]. The authors report that severe global stress is an independent risk factor for cardiovascular disease, even after adjustment for potential confounders such as obesity, diabetes, and hypertension.

Similarly, in 2016, the INTERSTROKE study, which included 26,919 participants, demonstrated that chronic stress is a risk factor for stroke with a population attributable risk of 15.1% for ischemic stroke [5].

Although cerebrovascular and cardiovascular diseases share a variety of common etiological pathways, we still need to distinguish between cerebrovascular events and MI. To take a case in point, whereas increased arousal may precipitate MI and sudden cardiac death, e.g., by inducing rupture of coronary atherosclerotic plaques or takotsubo cardiomypathy [6], there is no evidence that acute arousal constitutes an independent risk factor for stroke. Both INTERHEART and INTERSTROKE operationalized ‘stress’ as a multidimensional summary construct which covers stress at work and at home, financial stress, as well as major adverse life events in the preceding year. Apart from these psychological stressors, the two studies also showed that self-reported clinical depression was associated with higher risk of stroke [5] and MI [4], a remarkable epidemiological finding that has since been corroborated by several studies and meta-analyses [7–9].

Besides increasing the risk of CVD, psychosocial stress and depression exert a strong negative influence on functional outcome and recovery after stroke or myocardial infarction, an important clinical reality which is, unfortunately, frequently lost on health-care professionals.

## Biological effects of stress

**Fig. 1:**
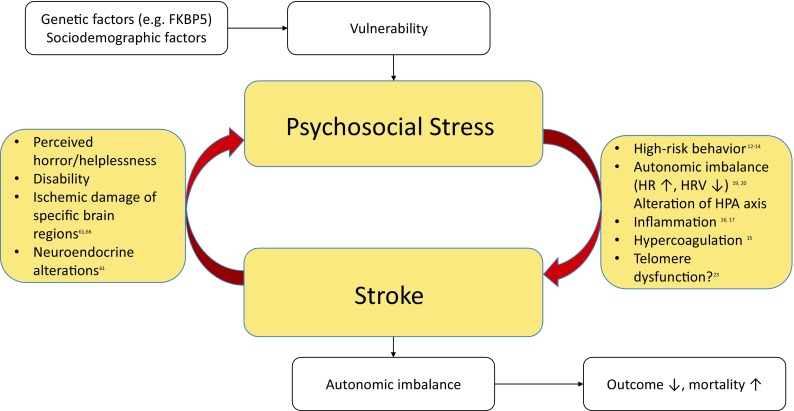
Psychosocial stress and stroke—a bidirectional relationship. *FKBP5* FK506 binding protein 5, *HR* heart rate, *HRV* heart rate variability, *HPA axis* hypothalamic-pituitary-adrenal axis

As illustrated in Fig. 1, there are various mechanisms by which adverse psychosocial conditions may confer an elevated cardiovascular risk. Broadly speaking, the physiological response to a stressful stimulus rests on two systems: the sympathetic nervous system (SNS) and the hypothalamic–pituitary–adrenal (HPA) axis. The sympathetic ‘fight and flight’ reaction entails an increased heart rate, vasoconstriction, bronchial dilation, and augmented muscular blood flow [10]. Activation of the HPA axis then induces a number of adaptive catabolic processes and suppresses both the reproductive and immune systems [10].

Although the two main stress systems play an indispensable role in protecting the organism from potential harm, their dysregulation and overactivation may also have deleterious effects. In particular, many psychiatric disorders are accompanied by neuroendocrine alterations: Major depression and anxiety disorders are associated with an exaggerated activation of the HPA axis [10], whereas patients with posttraumatic stress disorder (PTSD) typically display enhanced suppression of the negative glucocorticoid feedback loop and subsequent hypocortisolism [11]. Moreover, anxiety and excessively heightened arousal, common symptoms of psychiatric disorders, are associated with an increased tone of the SNS with overshooting catecholamine production, elevated heart rate (HR), and vasoconstriction.

Besides neuroendocrine alterations, behavioral factors play a fundamental role in the development of CVD: psychosocial stress and mental disorders favor high-risk lifestyle habits such as smoking, drinking, lack of exercise, and unhealthy eating [12–14], which, in turn, constitute risk factors for CVD. In addition, stress may cause a relevant hemostatic imbalance, as it promotes hypercoagulability in tandem with reducing fibrinolysis (for review, see [15]). An evolutionary explanation of increased clotting during acute emotional arousal could lie in the fact that, during much of human history, a moderate increase in coagulability in a sticky situation, so to speak, may have helped avert and minimize more serious harm. Most interestingly, the precise patterns of activation seem to differ between healthy subjects and CVD patients: In healthy controls, acute stress causes parallel stimulation of coagulation and fibrinolytic systems within a physiological range, whereas, in patients with CVD, the pro-coagulant reaction seems to predominate—possibly due to endothelial dysfunction and secondary insufficiency of fibrinolysis [15].

Systemic inflammatory and oxidative processes also contribute to stress-induced atherosclerosis. Psychosocial stress leads to increased plasma concentrations of cell adhesion factors and pro-inflammatory cytokines and lowers anti-inflammatory cytokines [16, 17], a mechanism that may promote CVD and immunological diseases. Acute inflammation may lead to a significant reduction of endothelium-dependent vasodilation, suggesting that endothelial dysfunction is one of the key mechanisms underlying stress-related cardiovascular risk [18]. Aside from the direct effects of inflammation on the vascular endothelium, centrally mediated changes in autonomic function may also be relevant [19, 20]. After induction of acute systemic inflammation by Typhim vaccination, an increase in the ratio of low-to-high frequency changes in heart rate (HR) variability was observed, suggesting an inflammatory modulation of the baroreflex with an increase in diastolic blood pressure [19].

Finally, psychosocial stress has been linked with elevated levels of reactive oxygen species (ROS), which may induce cellular injury, development of atherosclerotic plaques, and endothelial damage in the brain. Increased ROS concentrations have been demonstrated in humans after severe life experiences such as childhood abuse, divorce, or war, as well as in rodent models including predator exposure or maternal separation [21].

## Telomere biology

Telomeres are found on each end of eukaryotic chromosomes; they consist of repetitive hexamer DNA and multiple protein components, providing protection from degradation and maintaining chromosomal stability. Telomeres play a crucial rule in cellular senescence as they become shorter with each cell division, followed by a DNA damage response and, in the end, p53-mediated apoptosis [22]. The main function of the telomerase enzyme, a reverse transcriptase composed of a protein component (TERT) and an RNA component (TERC), is the elongation of telomeres and, hence, cellular regeneration and restoration.

Converging findings from animal and human studies suggest a close link between psychosocial stress and telomere biology. First, quite a number of studies have found that patients with psychiatric disorders such as major depression, ‘chronic mood disorders’ or bipolar disorder have shorter telomeres than healthy controls [23]. Similarly, prenatal and early life stress have been associated with shortened telomeres [23]. It has, therefore, been hypothesized that psychiatric patients are prone to accelerated telomere attrition, ultimately translating into premature senescence. However, a crucial question for which evidence has so far remained inconclusive concerns the relationship between telomerase activity and psychosocial stress: While some authors assume that decreased telomerase activity underlies telomere shortening in psychiatric disease, other studies have reported an increase in telomerase activity, suggesting a compensatory function of this enzyme [23].

The effect of stress on telomere biology and vascular senescence deserves special consideration as a potential mediator of increased cardiovascular risk. Although telomerase activity and telomere length have already been studied quite thoroughly in leukocytes, comparable knowledge of telomere biology in endothelial cells remains scant. Human aortic endothelial cells show telomere shortening with each cell division in vitro. Moreover, forced expression of TERT extends endothelial lifespan and attenuates phenotypic characteristics of cellular senescence such as reduced expression of endothelial NO synthase or increased binding of monocytes to endothelial cells. Together, these findings strongly suggest a crucial role of telomeres and TERT in vascular aging [24]. It has also been shown that inhibition of telomerase leads to a reduction in neointimal growth, a finding which implies that telomerase is also involved in the metabolism of the vascular smooth muscle cell [24]. Finally, in-vivo studies of human aortic endothelial cells revealed a reduction of telomere length with age [25].

Recent findings from animal studies provide further insight into the biological functions of telomerase. Mice null for TERT display a normal phenotype in the first generation, but their telomeres shorten in each successive generation [26]. Moreover, the later generations show a shorter lifespan and a diminished capacity to react to stresses such as wound healing and hematopoietic ablation [26]. After occlusion of the middle cerebral artery (MCAo), TERT knockout mice exhibit significantly larger infarct volumes and worse functional outcome along with elevated production of pro-inflammatory factors and a higher vulnerability to oxidative stress than littermate controls [27].

## Animal models of chronic stress and stroke

A variety of rodent stress models have been established which enable the assessment of the interplay between stress, depression, and vascular risk [28, 29]. The chronic stress model (CS) developed by Strekalova et al. [30] is a valid mouse model of anhedonia, one of the core symptoms of depression: Mice are exposed to a procedure consisting of several mild stressors (tail suspension, restraint, and predator-based stress) for 28 days. It has been shown that, after CS, mice consume less sucrose (a behavioral correlate of anhedonia) and show more anxiety-like behaviors [30]. In an adaptation of the procedure, we investigated the effects of chronic stress on endothelial function and stroke [28, 31]. Mice subjected to the CS procedure showed significantly enlarged brain lesions and impaired endothelium-dependent vasorelaxation after MCAo. The vascular effects of CS were at least partially mediated by glucocorticoid signaling as demonstrated by the fact that they were reversed by administration of glucocorticoid receptor (GR) antagonist mifepristone [31]. Interestingly, selective heart rate reduction (HRR) via the If channel inhibitor ivabradine also attenuated the detrimental effects of CS on endothelial function and thereby reduced the size of experimental infarcts after MCAo [28]. Our results are further corroborated by studies of apolipoprotein E^−/−^ mice showing decreased aortic atherosclerotic lesion size, reduction of oxidative stress, and improved endothelial function after ivabradine administration [32].

## FK506-binding protein 51

To this day, it has largely remained a mystery why some individuals are more susceptible to the adverse effects of psychosocial stress than others. Researchers in the field are grappling with how to unravel the complex interplay between genetic and epigenetic modulation, sociodemographic and environmental risk factors (e.g., childhood abuse and neglect, social support), and neurochemical pathways [33]. Recently, the FK506-binding protein 51 (FKBP5), a co-chaperone of the glucocorticoid receptor (GR) complex, has come into focus as a critical intracellular regulator of the stress response [34]. FKBP5 modulates the reactivity of the GR by inhibiting ligand binding and translocation of the receptor complex to the nucleus. As activation of the GR leads to increased FKBP5 transcription, this creates an ultra-fast negative feedback loop [35]. Emerging evidence points to the importance of FKBP5 in the development of stress-related mental disorders such as depression, anxiety disorders, and PTSD [36, 37]. Although the precise mechanisms remain to be elucidated, it appears safe to assume that epigenetic modulation and polymorphisms of FKBP5 contribute to the neuroendocrine alterations of the HPA system, which have been identified as a hallmark feature of stress-related mental illness. For example, it has been demonstrated that polymorphisms in FKBP5 are associated with increased recurrence of depressive episodes [36]. Moreover, several polymorphisms in the FKBP5 locus have been shown to significantly interact with the severity of child abuse to predict the development of PTSD symptoms in adulthood [37]. Furthermore, neuroimaging has revealed structural changes in emotion-processing brain areas in healthy individuals with experience of childhood abuse carrying the risk allele of the FKBP5 gene [38], suggesting one possible anatomical pathway for the increased vulnerability of those subjects towards stress. Findings from animal studies also support an important role of the co-chaperone in the stress response: While FKBP5^−/−^ mice do not differ from their littermates in behavior under basal conditions, they display increased resilience to stressful stimuli including more active coping behavior and reduced HPA axis reactivity [39]. In sum, these findings indicate that FKBP5 might be a very promising target for pharmacological manipulation in depression, PTSD, and anxiety disorders. Importantly, with regard to vascular disease, a study of healthy humans demonstrated interactions between adverse early life events and certain FKBP5 risk genotypes leading to an alteration of the heart rate response to mental stress [40], providing further evidence for the importance of gene–environment interactions in the pathogenesis of cardiovascular disease. Moreover, FKBP5 risk allele carriers exposed to a pre-learning stress condition display a significantly impaired performance in learning and memory compared to non-carriers [41], possibly due to a negative effect of certain FKBP5 polymorphisms on GR-mediated synaptic plasticity. The influence of FKBP5 polymorphisms on cognitive function seems to be even more pronounced in the elderly [42]. This finding is of particular interest for stroke patients, as learning deficits after stress exposure might have far-reaching consequences for neurological and psychological outcomes in the medium and long term.

Another important question concerns the possible interplay between FKBP5 polymorphisms and prior stressful/traumatic experiences in shaping the outcome of an incident stroke. Research into this issue is barely out of the gate, but may hold great promise in the related fields of personalized and preventive medicine.

## Heart rate (HR), stroke risk, and stroke outcome

While age, stroke severity, and blood pressure are widely established as predictors of stroke mortality, the value of HR as a prognostic indicator has so far been underappreciated. HR is an easily accessible measure on clinical examination. A relationship between HR and cardiovascular morbidity has been demonstrated in different patient populations such as patients with severe hypertension, heart failure, coronary artery disease, and even in healthy men [43–45]. A number of studies [46–48] have investigated the link between HR and ischemic stroke: in acute stroke patients without atrial fibrillation, higher HR on admission is associated with higher mortality, heart failure, and higher degree of dependence after 90 days [46, 49]. Similarly, a post hoc analysis in the PERFORM study population found that in patients with a recent noncardioembolic ischemic cerebral event, elevated HR is a negative prognostic factor and is associated with higher risk for MI [46, 49]. Since a rapid HR reflects, at least to a certain extent, increased levels of psychological stress [10], HR may be considered a surrogate marker of distress. Interestingly, elevated HR was also found to correlate with cognitive decline and poor outcome in patients after ischemic stroke [47]. Conversely, in experimental rodents, HR reduction increases brain capillary density and protects chronically stressed animals from oxidative damage [28].

One important issue still under debate is whether elevated HR is only a prognostic indicator for cardiovascular risk or if it is also a risk factor in its own right [48]. An increased HR is common in pre-existing cardiac disease and can reflect low levels of physical fitness, which makes it difficult to determine ‘cause and effect’. An increased HR spells increased oxygen consumption by the myocardium along with a decrease in coronary perfusion. Other mechanisms by which elevated HR may worsen cardiovascular outcome and increase mortality include arterial stiffness, disruption of atherosclerotic plaques, and cardiac arrhythmias. Initiated in 2014, the prospective SICFAIL cohort study aims to assess the prevalence of systolic dysfunction in stroke survivors, to identify key determinants of systolic dysfunction, and to chart its course after ischemic stroke [50].

Elevated HR variability and reduced HR variability indicate an imbalance of the autonomic nervous system—they can be understood as an exaggerated sympathetic response in conjunction with a suppression of vagal tone. Autonomic imbalance may have detrimental consequences including hypertension, increased blood clotting, and electrical cardiac instability [51]. Post-stroke patients, especially those who sustained a severe stroke [52], frequently display quite profound autonomic dysregulation [53], which has serious implications for life expectancy and quality of life. Apart from stroke severity, the exact site of the stroke in the brain is also of great importance for the development of autonomic imbalance. Specifically, studies in humans as well as in rodents have demonstrated the insula’s critical role in the regulation of the autonomic nervous system [54–56]. Lesions of the insular cortex, especially in the right hemisphere, are strongly associated with excess sympathetic and reduced parasympathetic tone [55], resulting in reduced HR variability, cardiac arrhythmias, and, ultimately, increased mortality [56].

There is considerable heterogeneity between stroke units in terms of the extent of cardiac monitoring. The prospective HEBRAS study [57] aims to clarify the association of stroke localization with autonomic dysbalance and cardiac dysfunction as well as the effects of reduced HR variability on long-term clinical outcomes. In patients with stable angina, HR reduction induced by ivabradine combined with beta-blockers was correlated with improved quality of life and a reduced number of angina attacks and nitrate consumption, suggesting that selective HR reduction may also be a promising new strategy in the treatment of stroke patients [58]. As we have described above, this approach is also supported by experimental research demonstrating that heart rate reduction improves aortic compliance in apolipoprotein E-deficient mice [28, 59] and protects chronically stressed animals from cerebral ischemia by improving endothelial function [28, 59].

## Psychiatric sequelae of stroke

Mood disorders and psychological distress are not only risk factors for CVD, they are also among the most common sequelae of brain ischemia (see Fig. 1). Post-stroke depression (PSD) occurs in approximately one-third of stroke survivors [60]. PSD is of enormous clinical significance as it is strongly associated with increased morbidity, mortality, and poorer functional outcome [61–63]. Far less well known is the disease entity of stroke-induced PTSD. However, a recent meta-analysis found that a staggering 23% of stroke and TIA patients show PTSD symptoms during the first year after the event [64]. Typically, patients suffering from PTSD present with symptoms of avoidance and intrusion, as well as alterations in arousal, mood, and cognition that last for at least 1 month. Similar to PSD, stroke-induced PTSD is associated with impaired functional outcome, poor medication adherence, recurrent stroke, and higher mortality [64, 65], which is why screening stroke patients for psychiatric symptoms should be an integral part of the diagnostic work-up.

Stroke is a highly disabling, potentially life-threatening illness that may provoke intense feelings of anxiety and fear. Unsurprisingly, stroke is frequently followed by long-lasting mental distress [60–62]. Moreover, ischemic damage of specific brain regions and stroke-related neuroendocrine alterations have also been causally implicated in the development of psychiatric morbidity after stroke [66]. Research conducted in experimental animals is beginning to further delineate the etiological roles of particular pathophysiological mechanisms triggered by brain ischemia such as neuroinflammation, disturbed cellular plasticity, neuroendocrine dysregulation, and neurodegeneration [66]. Recent work has especially highlighted the significance of delayed neurodegeneration [67]. In a model of mild transient brain ischemia, we found that delayed degeneration of dopaminergic neurons in the midbrain was associated with depressive-like behaviors [68]. Moreover, antidepressant treatment with a selective serotonin reuptake inhibitor (SSRI) initiated as late as 7 days after MCAo at least partly prevented degeneration of dopaminergic midbrain neurons [68]. These experimental findings dovetail with an emerging clinical literature suggesting that SSRI promote recovery after stroke [69].

## Conclusion

Substantial clinical and experimental evidence has accrued to indicate that psychosocial stress and mental disorders raise the risk for CVD. High-risk behaviors, neuroendocrine alterations, inflammatory changes, and oxidative stress are some of the underlying pathophysiological pathways mediating stress-induced endothelial dysfunction. Molecular factors such as the FKBP5 gene have been identified as critical modulators of the stress response. Moreover, exciting findings from human and animal studies have firmly linked mood disorders and psychological distress with perturbations in telomere biology and, thus, with cellular senescence. However, further studies are indispensable to bridge the gap between psychiatric research into gene–environment interactions and vascular outcomes after complex events such as stroke or MI.

HR is another parameter that reflects the intertwined effects of vascular morbidity and psychological distress. HR is emerging as a valuable predictor of stroke outcome and, possibly, a target for therapeutic intervention. In this article, our aim was to review major biological interfaces between stress and stroke, placing special emphasis on the bidirectionality of the relationship between these two. Comorbid mental disorders in stroke survivors may have far-reaching effects on recovery and outcome, including quality of life, recurrent ischemic events, medication adherence, and mortality. It would, therefore, be hard to overemphasize the importance of psychosocial factors in the prevention, treatment, and follow-up of patients with vascular disease.

## References

[1] Wilbert-Lampen U, Leistner D, Greven S, Pohl T, Sper S, Volker C (2008). Cardiovascular events during World Cup soccer. N Engl J Med.

[2] Mozaffarian D, Benjamin EJ, Go AS, Arnett DK, Blaha MJ, Cushman M (2016). Heart disease and stroke statistics-2016 Update: a report from the American heart association. Circulation.

[3] Haynes SG, Feinleib M, Kannel WB (1980). The relationship of psychosocial factors to coronary heart disease in the Framingham Study. III. Eight-year incidence of coronary heart disease. Am J Epidemiol.

[4] Rosengren A, Hawken S, Ounpuu S, Sliwa K, Zubaid M, Almahmeed WA (2004). Association of psychosocial risk factors with risk of acute myocardial infarction in 11119 cases and 13648 controls from 52 countries (the INTERHEART study): case-control study. Lancet.

[5] O’Donnell MJ, Chin SL, Rangarajan S, Xavier D, Liu L, Zhang H (2016). Global and regional effects of potentially modifiable risk factors associated with acute stroke in 32 countries (INTERSTROKE): a case-control study. Lancet.

[6] Esler M (2016). Mental stress and human cardiovascular disease. Neurosci Biobehav Rev.

[7] Pan A, Sun Q, Okereke OI, Rexrode KM, Hu FB (2011). Depression and risk of stroke morbidity and mortality: a meta-analysis and systematic review. JAMA.

[8] Wu Q, Kling JM (2016). Depression and the risk of myocardial infarction and coronary death: a meta-analysis of prospective cohort studies. Medicine (Baltimore).

[9] Nilsson FM, Kessing LV (2004). Increased risk of developing stroke for patients with major affective disorder–a registry study. Eur Arch Psychiatry Clin Neurosci.

[10] Charmandari E, Tsigos C, Chrousos G (2005). Endocrinology of the stress response. Annu Rev Physiol.

[11] Yehuda R (2000). Biology of posttraumatic stress disorder. J Clin Psychiatry.

[12] Slopen N, Kontos EZ, Ryff CD, Ayanian JZ, Albert MA, Williams DR (2013). Psychosocial stress and cigarette smoking persistence, cessation, and relapse over 9–10 years: a prospective study of middle-aged adults in the United States. Cancer Causes Control.

[13] Sims M, LipFord KJ, Patel N, Ford CD, Min YI, Wyatt SB (2017). Psychosocial factors and behaviors in African Americans: the jackson heart study. Am J Prev Med.

[14] Galea S, Nandi A, Vlahov D (2004). The social epidemiology of substance use. Epidemiol Rev.

[15] von Kanel R, Mills PJ, Fainman C, Dimsdale JE (2001). Effects of psychological stress and psychiatric disorders on blood coagulation and fibrinolysis: a biobehavioral pathway to coronary artery disease?. Psychosom Med.

[16] Heinz A, Hermann D, Smolka MN, Rieks M, Graf KJ, Pohlau D (2003). Effects of acute psychological stress on adhesion molecules, interleukins and sex hormones: implications for coronary heart disease. Psychopharmacology (Berl).

[17] Rohleder N (2014). Stimulation of systemic low-grade inflammation by psychosocial stress. Psychosom Med.

[18] Hingorani AD, Cross J, Kharbanda RK, Mullen MJ, Bhagat K, Taylor M (2000). Acute systemic inflammation impairs endothelium-dependent dilatation in humans. Circulation.

[19] Harrison NA, Cooper E, Voon V, Miles K, Critchley HD (2013). Central autonomic network mediates cardiovascular responses to acute inflammation: relevance to increased cardiovascular risk in depression?. Brain Behav Immun.

[20] Hering D, Lachowska K, Schlaich M (2015). Role of the sympathetic nervous system in stress-mediated cardiovascular disease. Curr Hypertens Rep.

[21] Schiavone S, Jaquet V, Trabace L, Krause KH (2013). Severe life stress and oxidative stress in the brain: from animal models to human pathology. Antioxid Redox Signal.

[22] Blasco MA (2007). Telomere length, stem cells and aging. Nat Chem Biol.

[23] Ridout SJ, Ridout KK, Kao HT, Carpenter LL, Philip NS, Tyrka AR (2015). Telomeres, early-life stress and mental illness. Adv Psychosom Med.

[24] Minamino T, Komuro I (2002). Role of telomere in endothelial dysfunction in atherosclerosis. Curr Opin Lipidol.

[25] Aviv H, Khan MY, Skurnick J, Okuda K, Kimura M, Gardner J (2001). Age dependent aneuploidy and telomere length of the human vascular endothelium. Atherosclerosis.

[26] Rudolph KL, Chang S, Lee HW, Blasco M, Gottlieb GJ, Greider C (1999). Longevity, stress response, and cancer in aging telomerase-deficient mice. Cell.

[27] Zhang B, Chen L, Swartz KR, Bruemmer D, Eum SY, Huang W (2010). Deficiency of telomerase activity aggravates the blood-brain barrier disruption and neuroinflammatory responses in a model of experimental stroke. J Neurosci Res.

[28] Custodis F, Gertz K, Balkaya M, Prinz V, Mathar I, Stamm C (2011). Heart rate contributes to the vascular effects of chronic mental stress: effects on endothelial function and ischemic brain injury in mice. Stroke.

[29] Kronenberg G, Kirste I, Inta D, Chourbaji S, Heuser I, Endres M (2009). Reduced hippocampal neurogenesis in the GR(+/-) genetic mouse model of depression. Eur Arch Psychiatry Clin Neurosci.

[30] Strekalova T, Spanagel R, Bartsch D, Henn FA, Gass P (2004). Stress-induced anhedonia in mice is associated with deficits in forced swimming and exploration. Neuropsychopharmacology.

[31] Balkaya M, Prinz V, Custodis F, Gertz K, Kronenberg G, Kroeber J (2011). Stress worsens endothelial function and ischemic stroke via glucocorticoids. Stroke.

[32] Custodis F, Baumhakel M, Schlimmer N, List F, Gensch C, Bohm M (2008). Heart rate reduction by ivabradine reduces oxidative stress, improves endothelial function, and prevents atherosclerosis in apolipoprotein E-deficient mice. Circulation.

[33] Heinz AJ, Beck A, Meyer-Lindenberg A, Sterzer P, Heinz A (2011). Cognitive and neurobiological mechanisms of alcohol-related aggression. Nat Rev Neurosci.

[34] Binder EB (2009). The role of FKBP5, a co-chaperone of the glucocorticoid receptor in the pathogenesis and therapy of affective and anxiety disorders. Psychoneuroendocrinology.

[35] Vermeer H, Hendriks-Stegeman BI, van der Burg B, van Buul-Offers SC, Jansen M (2003). Glucocorticoid-induced increase in lymphocytic FKBP51 messenger ribonucleic acid expression: a potential marker for glucocorticoid sensitivity, potency, and bioavailability. J Clin Endocrinol Metab.

[36] Binder EB, Salyakina D, Lichtner P, Wochnik GM, Ising M, Putz B (2004). Polymorphisms in FKBP5 are associated with increased recurrence of depressive episodes and rapid response to antidepressant treatment. Nat Genet.

[37] Binder EB, Bradley RG, Liu W, Epstein MP, Deveau TC, Mercer KB (2008). Association of FKBP5 polymorphisms and childhood abuse with risk of posttraumatic stress disorder symptoms in adults. JAMA.

[38] Grabe HJ, Wittfeld K, Van der Auwera S, Janowitz D, Hegenscheid K, Habes M (2016). Effect of the interaction between childhood abuse and rs1360780 of the FKBP5 gene on gray matter volume in a general population sample. Hum Brain Mapp.

[39] Touma C, Gassen NC, Herrmann L, Cheung-Flynn J, Bull DR, Ionescu IA (2011). FK506 binding protein 5 shapes stress responsiveness: modulation of neuroendocrine reactivity and coping behavior. Biol Psychiatry.

[40] Lovallo WR, Enoch MA, Acheson A, Cohoon AJ, Sorocco KH, Hodgkinson CA (2016). Early-life adversity interacts with FKBP5 genotypes: altered working memory and cardiac stress reactivity in the oklahoma family health patterns project. Neuropsychopharmacology.

[41] Zoladz PR, Dailey AM, Nagle HE, Fiely MK, Mosley BE, Brown CM (2016). FKBP5 polymorphisms influence pre-learning stress-induced alterations of learning and memory. Eur J Neurosci.

[42] Fujii T, Ota M, Hori H, Hattori K, Teraishi T, Matsuo J (2014). The common functional FKBP5 variant rs1360780 is associated with altered cognitive function in aged individuals. Sci Rep.

[43] Fox K, Borer JS, Camm AJ, Danchin N, Ferrari R, Lopez Sendon JL (2007). Resting heart rate in cardiovascular disease. J Am Coll Cardiol.

[44] Diaz A, Bourassa MG, Guertin MC, Tardif JC (2005). Long-term prognostic value of resting heart rate in patients with suspected or proven coronary artery disease. Eur Heart J.

[45] Jouven X, Escolano S, Celermajer D, Empana JP, Bingham A, Hermine O (2011). Heart rate and risk of cancer death in healthy men. PLoS One.

[46] Nolte CH, Erdur H, Grittner U, Schneider A, Piper SK, Scheitz JF (2016). Impact of heart rate on admission on mortality and morbidity in acute ischaemic stroke patients—results from VISTA. Eur J Neurol.

[47] Bohm M, Cotton D, Foster L, Custodis F, Laufs U, Sacco R (2012). Impact of resting heart rate on mortality, disability and cognitive decline in patients after ischaemic stroke. Eur Heart J.

[48] Bohm M, Reil JC, Deedwania P, Kim JB, Borer JS (2015). Resting heart rate: risk indicator and emerging risk factor in cardiovascular disease. Am J Med.

[49] Fox K, Bousser MG, Amarenco P, Chamorro A, Fisher M, Ford I (2013). Heart rate is a prognostic risk factor for myocardial infarction: a post hoc analysis in the PERFORM (prevention of cerebrovascular and cardiovascular events of ischemic origin with terutroban in patients with a history of ischemic stroke or transient ischemic attack) study population. Int J Cardiol.

[50] Bieber M, Kraft P, Ritter O, Frantz S, Heuschmann P, Kleinschnitz C (2015). SICFAIL—stroke induced cardiac failure in mice and men. Int J Stroke.

[51] Thayer JF, Yamamoto SS, Brosschot JF (2010). The relationship of autonomic imbalance, heart rate variability and cardiovascular disease risk factors. Int J Cardiol.

[52] Hilz MJ, Moeller S, Akhundova A, Marthol H, Pauli E, De Fina P (2011). High NIHSS values predict impairment of cardiovascular autonomic control. Stroke.

[53] Dutsch M, Burger M, Dorfler C, Schwab S, Hilz MJ (2007). Cardiovascular autonomic function in poststroke patients. Neurology.

[54] Oppenheimer SM, Cechetto DF (1990). Cardiac chronotropic organization of the rat insular cortex. Brain Res.

[55] Meyer S, Strittmatter M, Fischer C, Georg T, Schmitz B (2004). Lateralization in autonomic dysfunction in ischemic stroke involving the insular cortex. Neuroreport.

[56] Tokgozoglu SL, Batur MK, Topcuoglu MA, Saribas O, Kes S, Oto A (1999). Effects of stroke localization on cardiac autonomic balance and sudden death. Stroke.

[57] Haeusler KG, Grittner U, Fiebach JB, Endres M, Krause T, Nolte CH (2015). Heart and brain interfaces in acute ischemic stroke (HEBRAS)—rationale and design of a prospective oberservational cohort study. BMC Neurol.

[58] Werdan K, Ebelt H, Nuding S, Hopfner F, Hack G, Muller-Werdan U (2012). Ivabradine in combination with beta-blocker improves symptoms and quality of life in patients with stable angina pectoris: results from the ADDITIONS study. Clin Res Cardiol.

[59] Custodis F, Fries P, Muller A, Stamm C, Grube M, Kroemer HK (2012). Heart rate reduction by ivabradine improves aortic compliance in apolipoprotein E-deficient mice. J Vasc Res.

[60] Hackett ML, Pickles K (2014). Part I: frequency of depression after stroke: an updated systematic review and meta-analysis of observational studies. Int J Stroke.

[61] Loubinoux I, Kronenberg G, Endres M, Schumann-Bard P, Freret T, Filipkowski RK (2012). Post-stroke depression: mechanisms, translation and therapy. J Cell Mol Med.

[62] Towfighi A, Ovbiagele B, El Husseini N, Hackett ML, Jorge RE, Kissela BM (2016). Poststroke depression: a scientific statement for healthcare professionals from the American heart association/American stroke association. Stroke.

[63] Hellmann-Regen J, Piber D, Hinkelmann K, Gold SM, Heesen C, Spitzer C (2013). Depressive syndromes in neurological disorders. Eur Arch Psychiatry Clin Neurosci.

[64] Edmondson D, Richardson S, Fausett JK, Falzon L, Howard VJ, Kronish IM (2013). Prevalence of PTSD in survivors of stroke and transient ischemic attack: a meta-analytic review. PLoS One.

[65] Garton AL, Sisti JA, Gupta VP, Cristophe BR, Connolly ES (2017). Poststroke post-traumatic stress disorder: a review. Stroke.

[66] Kronenberg G, Gertz K, Heinz A, Endres M (2014). Of mice and men: modelling post-stroke depression experimentally. Br J Pharmacol.

[67] Baron JC, Yamauchi H, Fujioka M, Endres M (2014). Selective neuronal loss in ischemic stroke and cerebrovascular disease. J Cereb Blood Flow Metab.

[68] Kronenberg G, Balkaya M, Prinz V, Gertz K, Ji S, Kirste I (2012). Exofocal dopaminergic degeneration as antidepressant target in mouse model of poststroke depression. Biol Psychiatry.

[69] Mead GE, Hsieh CF, Lee R, Kutlubaev MA, Claxton A, Hankey GJ (2012). Selective serotonin reuptake inhibitors (SSRIs) for stroke recovery. Cochrane Database Syst Rev.

